# Do computer use, TV viewing, and the presence of the media in the bedroom predict school-aged children’s sleep habits in a longitudinal study?

**DOI:** 10.1186/1471-2458-13-684

**Published:** 2013-07-26

**Authors:** Teija Nuutinen, Carola Ray, Eva Roos

**Affiliations:** 1 Folkhälsan Research Center, Paasikivenkatu 4, 00250 Helsinki, Finland; 2 Department of Public Health, Hjelt institute, University of Helsinki, Helsinki, Finland

**Keywords:** Child, Sleep, Television viewing, Computer use, Longitudinal

## Abstract

**Background:**

Electronic media use is becoming an increasingly important part of life for today’s school-aged children. At the same time, concern of children’s sleep habits has arisen, and cross-sectional studies have shown that electronic media use is associated with short sleep duration and sleep disturbances. The purpose of this longitudinal study was to investigate whether baseline electronic media use and media presence in a child’s bedroom predicted sleep habits as well as changes in these sleep habits 18 months later among 10- to 11-year-old children in Finland.

**Methods:**

The school-aged children (n=353, 51% girls) from 27 schools answered a questionnaire in 2006 and again 2008 in the Helsinki region of Finland. Electronic media use was measured by computer use and TV viewing. Media presence in a child’s bedroom means the presence of a TV or a computer in a child’s bedroom. Sleep habits were measured by bedtimes on school days and at the weekend days, sleep duration, discrepancy of bedtimes, and discrepancy of sleep duration between school days and weekends. Linear regression analyses were used to examine whether electronic media use and media presence predicted sleep habits with adjustments for grade, family structure, and baseline sleep. Gender differences were also examined.

**Results:**

The children used a computer for one hour per day and watched TV over one hour a day in 2006. They slept over nine hours on school days and over ten hours at the weekends in 2008. Computer use and television viewing predicted significantly shorter sleep duration (p<0.001, p<0.05 respectively) and later bedtimes (p<0.001, p<0.01, respectively). Computer use also predicted unfavourable changes in sleep duration (p<0.001) and bedtimes on school days (p<0.001) and weekends (p<0.01). Among boys, media presence in the bedroom predicted poorer sleep habits and irregularity of sleep habits.

**Conclusions:**

Computer use, TV viewing, and the presence of media in children’s bedrooms may reduce sleep duration, and delay bedtimes.

## Background

Sleep is crucial for daily functioning as well as mental and physical health. In school-aged children poor sleep quality and sleep habits are associated with learning difficulties, lower school achievement, and memory problems [1]. When children grow older sleep duration tends to decrease [2]. Decreasing sleep duration is an unfavourable change during early youth, because physiological changes related to puberty may even increase the need for sleep [3]. Along with a reduction in sleep duration, daytime sleepiness also tends to increase [4].

Many factors may reduce sleep duration and delay bedtimes, and one such potential factor may be the increased use of computers and TV viewing among school-aged children. Computer use or TV viewing may simply displace sleep, thus reducing its duration. An indirect pathway may also explain the association between computer use, TV viewing and sleep; for example, computer use or TV viewing may displace physical activity [5], which is known to promote good quality sleep [6]. Electronic media use may increase physiological and mental arousal, which makes it difficult to fall asleep [7]. In addition, computer use or TV viewing may actually affect the sleep architecture by, for example, decreasing slow-wave sleep, REM-sleep, and sleep efficiency [7,8], or the bright light of a television or computer screen may suppress melatonin secretion, which in turn may delay the onset of sleep [9].

Many cross-sectional studies have reported associations between computer use, TV viewing, and sleep habits among school-aged children [10-12]. Research has shown that the total amount of television viewed or computer use a day is associated with reduced sleep duration, later bedtimes and wake-up times [10,12], and irregular sleep-wake patterns [13]. A recent study also showed that computer use or TV viewing two or more hours a day, and having a bedroom TV were risk factors for short sleep duration among school-aged children in Sweden [14]. The presence of media, that is, a computer or a TV, in children’s bedrooms is associated with later bedtimes and wake-up times, shorter sleep duration [15], and self-perceived sleep problems [12,16]. However, not all of the studies have shown significant associations, for instance, between television viewing and reduced sleep duration [17-19]. To obtain a more comprehensive picture, more longitudinal studies are needed. To our knowledge, no longitudinal studies have examined the association between electronic media use and sleep habits. Prospective studies have only examined sleep problems, and electronic media use had increased the risk of sleep problems in these studies [20-22].

The first aim of this study was to examine whether baseline computer use, television viewing as well as media presence in a child’s bedroom predicted sleep habits such as bedtimes on school days and at weekends, sleep duration, and irregular sleep habits among school-aged children in an eighteen-month longitudinal study. The second was to examine whether these above-mentioned determinants also predicted changes in these sleep habits. The third was to examine whether the examined associations varied by gender.

## Methods

The study was conducted as part of the school project*, The Hälsoverkstaden “Health workshop”*, which endeavoured to examine health behaviours and determinants related to health behaviours among 10- to 11-year-old (4^th^ and 5^th^ graders) children. Baseline measurements took place in the autumn of 2006 and eighteen-months later in the spring of 2008. Forty-four Swedish-speaking schools in the Helsinki region of Finland were invited to participate and the decision to take part was made by the principals in 27 schools. All 4^th^ and 5^th^ graders in the 27 schools were invited to participate in the study. The response rates were 74% (n=858) in 2006, and 79% of the baseline participants (n=677) also participated in 2008. An intervention was made in half of the schools (n=13). Schools were randomly chosen to be either intervention or control schools. In our analyses, we only included those pupils from control schools (n=353) to avoid the intervention having a any effect on the results. At the baseline in 2006 51% (n = 181, 52% girls n = 94) of the children were at grade 4 in the control schools, the rest being at grade 5 (n = 172, 51% girls n = 87). In 2008, 52% (n = 183, 52% girls n = 95) were at grade 5 and the rest were at grade 6 (n = 170, 50% girls n = 85).

The pupils completed a questionnaire about health behaviours in a supervised classroom situation where a member of the research staff was continuously present. Being absent from school on the day of the data collection or having moved to an other school were the main reasons for not participating in the follow-up.

*The Hälsoverkstaden, “Health workshop”,* project was approved by the Ethics Committee of the Department of Public Health, University of Helsinki, in the spring of 2006. Parents gave their written consent for their children to take part and the children also gave their own consent. Participation was voluntary, and the children and the parents were informed that they were free to withdraw at any phase of the study.

### Measures

Sleep habits were assessed as bedtimes and wake-up times on school days and at weekends with the questions: “When do you usually go to bed if the next morning is a school day/is not a school day?” The alternative answers for bedtimes and wake-up times were listed using half-hour intervals. For school days, the alternative answers for bedtime ranged from 20.00 to 00.30 or later and for weekends the alternative answers ranged from 20.30 to 1.00 or later. For school days the alternative answers for wake-up time ranged from 6.00 to 9.00 and for weekends the alternative answers ranged from 6.30 to 11.00. Sleep duration, separately for school days and weekends, was calculated from bedtimes and wake-up times using the following formula: 24-(bedtime in 24 h hour clock time + wake-up time in 24 h clock time). Irregular sleep habits were assessed with two variables: (1) discrepancy of bedtime between school days and weekends (bedtime on weekends - bedtime on school days) and (2) discrepancy in sleep duration between school days and weekends (weekend sleep duration - school day sleep duration). Bedtimes, wake-up times, sleep duration and irregular sleep habits were treated as continuous variables.

Television viewing and computer use were enquired about as follows: “How many hours daily do you usually watch television, videos or DVDs?” and “How many hours daily do you usually use a computer or play games with a console?” The seven alternative answers ranged from “not at all” to “about 5 hours or more”, which were scored according to time spent: 0, 0.5, 1, 2, 3, 4, and 5 hours. TV viewing and computer use was only applied to school days. Television viewing and computer use were treated as continuous variables.

The presence of media in children’s bedrooms was enquired about using two questions: “Do you have a television in your own bedroom?” and “Do you have a computer or game console in your own bedroom?” The alternative answers were yes and no, and media presence was treated as a dichotomous variable in the analyses.

The number of missing values of examined variables varied between 0 and 26. There were most missing values for having a computer in the bedroom. There were no missing values for bedtimes and wake-up times at weekends, and sleep duration at weekends in 2008.

Confounding variables included grade (4^th^ and 5^th^), gender, and family structure (living with both parents, not living with both parents). Grade was used to adjust for age because grade is a good indicator of age in Finland. When children begin school in Finland, they are all born in the same calendar year and only a few repeat a class. The family structure was chosen as a confounder since it seems that the children in single-parent families use electronic media more than children living with both their mothers and fathers [23], and bedtime routines are more common in two-parent families than in single-parent ones [24].

### Statistical methods

Descriptive statistics are represented as means and standard deviations and as percentages in the case of categorical variables. Differences in sleep habits, TV viewing, and the presence of a TV and a computer in children’s bedrooms between genders were examined by an independent-samples t-test and a Chi-square test. Differences in sleep habits between in 2006 and in 2008 were examined by a paired sample t-test.

Linear regression analyses were performed to examine whether baseline computer use (h/day), television viewing (h/day), and baseline TV and computer presence in children’s bedrooms predicted sleep habits eighteen-months later. Separate models were conducted for all the different media variables. Model 1 (linear regression) was adjusted for the children’s gender, grade, and family structure. Further, model 2 was adjusted for the baseline corresponding sleep variable to determine factors related to changes in sleep habits. To examine whether computer use, TV viewing, and media presence in the bedroom had a different effect on sleep by gender, we explored interactions by including an interaction term in the general linear models. The interaction term always included gender and one of the media variables. The regression analyses were performed separately for girls and boys with the variables indicating statistically significant interaction.

We chose 0.05 as a statistically significant level. For the interactions the statistically significant level was 0.1 [25]. The analyses were carried out using Predictive Analytics SoftWare (PASW) Statistics release version 18.0 for Windows (SPSS Inc. 2010).

## Results

Table 1 illustrates sleep habits in 2006 and in 2008 and changes in sleep habits from 2006 to 2008 separated for girls and boys. Among both girls and boys sleep duration decreased during the eighteen-months on school days, but not at weekends. Bedtimes at weekends were later and sleep duration was longer than on school days. Girls slept longer on weekends and they had a greater discrepancy in sleep duration between school days and weekends than boys in both study years. Discrepancy in sleep duration increased among boys and girls between 2006 and 2008. In 2008, sleep duration on school days was shorter among girls than among boys. In 2008, bedtimes were later on school days and at weekends, and wake-up times on school days were earlier than in 2006. The discrepancy in bedtimes between school days and weekends increased between the study years. In 2008, boys had a greater discrepancy in bedtime between school days and weekends than girls.

**Table 1:** Sleep habits shown as means (hh:mm/day) and standard deviations (SD) among Finnish school-aged children (n = 337–353)†

	**2006**		**2008**		**∆ 2006–2008**	
	4th and 5th graders		5th and 6th graders			
Girls (n = 177–181) †	mean	SD	mean	SD	mean	SD
Bedtime school day	21:29	0:37	21:48	0:37	0:23***	0:36
Wake-up time school day	7:10	0:26	7:01	0:25	−0:10***	0:30
Bedtime weekend	22:18	0:48	22:44	0:48	0:26***	0:47
Wake-up time weekend	8:42	0:53	9:08	0:59	0:27***	0:52
Discrepancy of bedtimes	0:53	0:35	0:56	0:38	0:04	0:42
Sleep duration school day	9:48	0:40	9:13	0:43	−0:32***	0:46
Sleep duration weekend	10:24	0:57	10:24	1:00	0:01	1:04
Discrepancy in sleep duration	0:39	0:58	1:12	1:00	0:33***	1:07
Boys (n = 159–172) †						
Bedtime school day	21:24	0:41	21:41	0:39	0:18***	0:39
Wake-up time school day	7:06	0:30	7:07	0:25	0:00	0:35
Bedtime weekend	22:24	0:50	22:51	0:59	0:27***	0:48
Wake-up time weekend	8:27	1:08	8:54	1:10	0:27***	1:01
Discrepancy of bedtimes	1:01	0:41	1:11	0:47	0:09*	0:47
Sleep duration school day	9:42	0:48	9:25	0:44	−0:18***	0:48
Sleep duration weekend	10:03	1:12	10:03	1:08	0:00	1:15
Discrepancy in sleep duration	0:20	1:13	0:37	1:08	0:18**	1:13
All (n = 337–353) †						
Bedtime school day	21:25	0:39	21:48	0:38	0:20***	0:37
Wake-up time school day	7:08	0:28	7:03‡	0:25	−0:05**	0:32
Bedtime weekend	22:21	0:49	22:48	0:54	0:26***	0:47
Wake-up time weekend	8:35‡	1:01	9:01‡	1:05	0:27***	0:57
Discrepancy of bedtimes	0:57	0:38	1:04‡‡	0:44	0:06*	0:44
Sleep duration school day	9:43	0:44	9:19‡‡	0:44	−0:25***	0:47
Sleep duration weekend	10:14‡‡	1:05	10:14‡‡	1:05	0:00	1:10
Discrepancy in sleep duration	0:30‡‡	1:06	0:55‡‡‡	1:06	0:26***	1:10

† Range of respondents in the analyses.

Difference between study years *** ≤0.001, **≤0.01 *≤0.05, paired sample t-test.

Difference between gender within years ‡‡‡≤0.001, ‡‡≤0.01, ‡≤0.05, independent-samples t-test.

Table 2 describes computer use, television viewing, and media presence in 2006. Boys were more likely to have a television and a computer in their bedrooms than girls, and boys used computers more than girls (Table 2).

**Table 2:** Media presence in bedrooms shown as percentages, and computer use and TV viewing shown as means (hh:mm/day) and standard deviations (SD) (hh:mm) among Finnish schoolchildren in 2006 (n = 327–353)†

	**% or mean**	**SD**	Difference between genders, p-values*
**Girls** (n = 163–181)†			
Computer in bedroom yes %	28		
Television in bedroom yes %	29		
**Boys** (n = 161–172)†			
Computer in bedroom yes %	57		
Television in bedroom yes %	48		
**All** (n = 327–353)†			
Computer in bedroom yes %	43		≤0.001
Television in bedroom yes %	39		≤0.001
**Girls** (n = 178–181)†			
Computer use (hh:mm/day)	0:49	0:52	
Television viewing (hh:mm/day)	1:18	0:58	
**Boys** (n = 169–172)†			
Computer use (hh:mm/day)	1:11	1:02	
Television viewing (hh:mm/day)	1:15	0:56	
**All** (n = 348–353)†			
Computer use (hh:mm/day)	0:59	0:58	≤0.001
Television viewing (hh:mm/day)	1:17	0:57	

† Range of respondents in the analyses.

*Chi-square test for media presence and independent samples t-test for computer use and TV viewing.

Baseline computer use and television viewing predicted shorter sleep duration on school days eighteen-months later (Model 1 in Table 3). Computer use and television viewing also predicted later bedtimes on school days. Computer use and having a computer in the bedroom also predicted later bedtimes at weekends.

**Table 3:** The associations examined by linear regression analyses between baseline computer use (hh:mm/day), TV viewing (hh:mm/day), media presence in bedroom (yes/no) and sleep habits (hh:mm/day) eighteen-months later among Finnish school-aged children (n = 327–353)†

	**Model 1**		**Model 2**	
	**B-coefficient**	**95% CI**	**B-coefficient**	**95% CI**
	Computer in bedroom			
Sleep duration school days	−0.15	−0.31-0.01	−0.11	−0.27-0.04
Sleep duration weekends	−0.01	−0.25-0.24	−0.04	−0.26-0.19
Bedtime school day	0.13	−0.01-0.27	0.09	−0.04-0.21
Bedtime weekend	0.25	0.05-0.45*	0.16	−0.01-0.33
Discrepancy of bedtime school days and at weekends	0.15	−0.02-0.32	0.12	−0.03-0.27
Discrepancy in sleep duration school and at weekends	0.15	−0.11-0.40	0.07	−0.17-0.3
	Television in bedroom			
Sleep duration school day	−0.09	−0.26-0.07	−0.09	−0.24-0.07
Sleep duration weekends	−0.14	−0.10-0.39	0.11	−0.12-0.34
Bedtime school day	0.12	−0.02-0.26	0.10	−0.02-0.23
Bedtime weekends	0.18	−0.01-0.38	0.11	−0.06-0.27
Discrepancy of bedtime school day and at weekends	0.08	−0.08-0.24	−0.02	−0.13-0.16
Discrepancy in sleep duration school day and at weekends	0.24	−0.01-0.48	0.19	−0.04-0.42
	Computer use			
Sleep duration school day	−0.18	−0.26-(−)0.10***	−0.14	−0.22-(−)0.07***
Sleep duration weekends	−0.09	−0.21-0.032	−0.09	−0.20-0.02
Bedtime school day	0.19	0.13-0.26***	0.14	0.08-0.20***
Bedtime weekends	0.19	0.09-0.29***	0.12	0.04-0.20**
Discrepancy of bedtime school day and at weekends	−0.004	−0.08-0.07	0.004	−0.07-0.07
Discrepancy in sleep duration school day and at weekends	0.10	−0.02-0.22	0.06	−0.05-0.17
	Television viewing			
Sleep duration school day	−0.09	−0.17-(−)0.01*	−0.07	−0.14-0.01
Sleep duration weekends	−0.01	−0.13-0.11	−0.01	−0.12-0.11
Bedtime school day	0.10	0.04-0.17**	0.07	0.01-0.13*
Bedtime weekends	0.10	−0.001-0.20	0.04	−0.04-0.13
Discrepancy of bedtime school day and at weekends	−0.01	−0.09-0.07	−0.02	−0.09-0.05
Discrepancy in sleep duration school day and at weekends	0.09	−0.03-0.21	0.08	−0.04-0.19

† Range of respondents in the analyses.

*CI* confidence interval.

Model 1: Adjusted for gender, grade, nuclear family.

Model 2: Adjusted for gender, grade, nuclear family, and corresponding sleep variable 2006.

*<0.05, **<0.01, ***<0.001.

All independent variables are included separately in the models.

Computer use and television viewing predicted a greater decrease in sleep duration on school days, and a greater increase in bedtimes on school days. Computer use also predicted a greater increase in bedtimes on weekends. (Model 2 in Table 3).

### Gender-stratified analyses

Interactions existed between gender, the presence of media in children’s bedrooms, and television viewing. Interactions were found between gender and computer in the bedroom and bedtime on school days (p=0.041), gender and computer in the bedroom and bedtime discrepancy (p=0.093), gender and computer in the bedroom and bedtime on weekends (p=0.006), gender and television in the bedroom and bedtime on weekends (p=0.036), gender and television in the bedroom and bedtime on school days (p=0.054), gender and television in the bedroom, and sleep duration on weekends (p=0.017), gender and television in the bedroom and discrepancy in sleep duration (p=0.083), and gender and television viewing and bedtime discrepancy (p=0.081). Regression analyses were performed separately for girls and boys using the independent variables that indicated interaction. A television in the bedroom predicted later bedtimes on school days and at weekends and a greater increase of bedtime on school days and weekends among boys but not among girls. A television in the bedroom predicted longer sleep duration on weekends and a greater increase in sleep duration on weekends among girls. A television in the bedroom also predicted greater discrepancy in sleep duration between school days and weekends and a greater increase in discrepancy in sleep duration among girls. A computer in the bedroom predicted later bedtime on school days and at weekends, a greater increase of bedtime on school days and weekends, and a greater bedtime discrepancy between school days and weekends among boys (Table 4).

**Table 4:** Linear regression analyses separately for boys (n = 161–172)† and girls (n = 163–181)† for variables indicating interaction with gender

	**Boys**		**Girls**	
	**Model 1**	**Model 2**	**Model 1**	**Model 2**
	**B-coefficient [95% CI]**	**B-coefficient [95% CI]**	**B-coefficient [95% CI]**	**B-coefficient [95% CI]**
	Television in the bedroom (yes/no)		Television in the bedroom (yes/no)	
Bedtime school day (hh:mm)	0.25 [0.05-0.44*]	0.26 [0.09-0.43**]	−0.015 [−0.21-0.18]	−0.06 [−0.23-0.11]
Bedtime weekends (hh:mm)	0.38 [0.09-0.66**]	0.30 [0.07-0.53*]	−0.034 [−0.30-0.23]	−0.10 [−0.33-0.12]
Sleep duration weekends (hh:mm/day)	−0.12 [−0.47-0.24]	−0.197 [−0.53-0.14]	0.44 [0.10-0.77**]	0.45 [0.14-0.76**]
Discrepancy in sleep duration (hh:mm)	0.05 [−0.31-0.42]	0.011 [−0.32-0.34]	0.45 [0.12-0.78**]	0.38 [0.06-0.70*]
	Computer in the bedroom (yes/no)		Computer in the bedroom (yes/no)	
Bedtime school day (hh:mm)	0.26 [0.07-0.44**]	0.20 [0.03-0.37*]	−0.02 [−0.23-0.18]	−0.05 [−0.23-0.14]
Bedtime weekends (hh:mm)	0.49 [0.21-0.77***]	0.32 [0.10-0.56**]	−0.04 [−0.32-0.24]	−0.05 [−0.30-0.19]
Bedtime discrepancy (hh:mm)	0.28 [0.03-0.52*]	0.20 [−0.02-0.41]	−0.01 [−0.24-0.23]	0.02 [−0.20-0.24]
	Television viewing (hh:mm/day)		Television viewing (hh:mm/day)	
Bedtime discrepancy (hh:mm)	−0.09 [−0.22-0.04]	−0.06 [−0.18-0.05]	0.06 [−0.04-0.17]	0.02 [−0.08-0.12]

† Range of respondents in the analyses.

*CI* confidence interval.

Model 1: adjusted for grade and family structure.

Model 2: adjusted for grade, family structure and corresponding sleep variable 2006.

*<0.05, **<0.01, ***<0.001.

All independent variables are included separately in the models.

## Discussion

Our main findings were that computer use and television viewing predicted shorter sleep duration and later bedtimes. A computer in the bedroom predicted later bedtimes on weekends. The more children used a computer or watched a TV the greater was the decrease in sleep duration and the delay in bedtime eighteen-months later. Gender differences existed in the associations between media presence in the bedroom, bedtimes, discrepancy in bedtimes and sleep duration between school days and weekends, and sleep duration on weekends.

Since no longitudinal studies exist in this research field, these results provide new information that computer use and television viewing were associated with sleep habits eighteen-months later. Many cross-sectional studies are in line with our findings that TV viewing, computer use, and media presence in a child’s bedroom are related to shorter sleep duration [10,14] and later bedtimes [12]. For example, television viewing was associated with short sleep duration on school days among 5- to 12-year-olds and later bedtimes and wake-up times among 12- to 19-year-olds in the U.S. Computer use was also associated with phase-delayed sleep schedules, but not total sleep time (10). A recent study also found that having a bedroom TV, spending more than 2 hours a day at a TV or computer were associated with short sleep duration among school-aged children in Sweden [14]. Similar findings have also been reported in Belgium among older children [12]. Van den Bulck (2004) found that media presence in bedroom, TV viewing and computer use were associated with later bedtimes among 13- to 16-year-old adolescents [12]. Whether a child has a TV or a computer in his/her bedroom also varies by country. In our study nearly half had a bedroom TV and a third had a computer. In Sweden, lower rates of bedroom TV have been reported than in our study, one third of 4^th^ graders had one [14]. It seems that bedroom TV is more prevalent in the U.S. than in Europe. However, the presence of a computer in the bedroom seems to be as common as in the U.S. as in our sample. With eight- to 18-year-olds, 71% had a TV in their bedroom and 36% had a computer in their bedroom in the U.S. [26]. In our study, computer use was more strongly associated with later bedtimes and shorter sleep duration than TV viewing. Computer use is usually more active than TV viewing, and computer use may stimulate the brain more and increase physiological arousal more than TV viewing [9].

Our findings indicate that a media presence in the bedroom may affect sleep differently between genders. When girls and boys were analyzed separately, we found significant associations with irregular sleep habits. A television and a computer in the bedroom predicted later bedtimes on school days and at weekends and a computer in the bedroom also predicted a greater discrepancy in bedtimes between school days and weekends among boys but not among girls. Girls who had a television in their bedroom slept even longer on weekends than girls who had no television in their bedroom. The discrepancy in sleep duration between school days and weekends was also greater among girls who had a television in their bedroom than among girls who did not. Among boys, there was no association between having a television in the bedroom, sleep duration on weekends and discrepancy in sleep duration. One of the interesting findings was that having a computer in the bedroom was associated with poorer sleep habits only among boys. Another Finnish study has shown that intensive computer use constitutes a risk in terms of poor health, particularly for boys [27]. It is unclear why these gender differences exist, but boys and girls tend to use computers differently; boys tend to play games and spend more time in front of the computer whereas girls use computers more for communicating with friends [27]. Gender differences may exist in the associations between electronic media use and sleep habits because pubertal status is associated with sleep differently by gender. It has been shown that pubertal development increased sleep problems among girls, but not among boys. Pubertal development and sleep duration were negatively associated in both sexes [28].

Certainly, many other factors related to electronic media use may also influence sleep habits and changes in sleep habits. Older children tend to go bed later than their younger counter parts, and sleep duration decreases [2]. In addition, parental supervision may decline concerning electronic media use and bedtimes when children grow older. In 2006, the children in our study were 10- to 11-year-olds and in 2008 11- to 12-year-olds. It is important to take the role of these age-related changes in sleep and changes in parental supervision in account when evaluating our results concerning changes in sleep habits, although the children’s school grade (indicating age) was adjusted. In addition, computer use and TV viewing are likely accompanied by low physical activity [5], an unhealthy diet [29], and overweight [30], all features of a sedentary lifestyle. All these factors may, independently or together, be related to sleep. We also did regression analyses where we adjusted for physical activity, but it did not change the results, and therefore we did not include it in the final analyses.

This study includes a number of strengths. The longitudinal design with a large sample size provides valuable information, particularly since rather few longitudinal studies exist in this research area. To our knowledge this is the first longitudinal study to examine the effects of computer use on sleep habits. We also examined several associations between computer use, television viewing, media presence in children’s bedrooms and sleep habits. Another strength that in this longitudinal study we examined interactions between gender and media variables related to sleep habits, and we found interesting gender differences when analyzing boys and girls separately. To our knowledge this was the first study that examined whether there are gender differences in the associations between media and sleep habits among school-aged children in a longitudinal study.

This study does have some limitations that should be considered. The sample is selected because it consisted of pupils from Swedish-speaking schools representing a language minority in the Helsinki region in Finland. In addition, the proportion highly educated parents was higher than the national average. With a more heterogeneous sample, more variation would probably have existed in computer use, television viewing and sleep variables, and the associations would have been stronger. Another limitation is the assessment of television viewing, computer use, media presence in the bedroom, and sleep habits by self-report questionnaires. However, bedtimes and wake-up times assessed by questionnaire seems to be a valid method to assess bedtimes and sleep duration compared to actigraphy measurements and sleep diaries [31]. Research also suggests that 8- to 11-year-old children can report their health quite reliably, particularly if the questions have been developed specifically for children [32]. Similar kinds of measurement of computer use and television viewing has achieved an adequate degree of reliability and validity in previous studies [33,34]. In future studies more objective measurements of sleep could be used (like actigraphs) together with questionnaires and sleep diaries. In addition, the actigraph could provide more detailed information on electronic media use together with media diary. To clarify current findings, studies with a longer follow-up, along with multiple follow-ups and more objective measurements, are needed.

## Conclusions

In this longitudinal study, computer use and television viewing predicted later bedtimes, shorter sleep duration, and changes in these sleep habits. A media presence in the bedroom was also related to irregular sleep habits: a television and a computer in the bedroom among boys, and a television in the bedroom among girls. Thus, a computer in the bedroom predicted irregular sleep habits only among boys. Parents, teachers and health care providers should be aware that television viewing and computer use may have an adverse impact on sleep, which in turn may lead to daytime tiredness, attention and behavioral problems as well as increased health risks in the long run. Electronic media devices should not be placed in a child’s bedroom. The screening of media use habits, as well as sleep habits, should therefore be included in health behavior screening. Special attention should be paid to children’s media use during weekdays, since our estimates were stronger for sleep duration on school days than at weekends.

## Competing interests

The authors declare that they have no competing interests.

## Authors’ contributions

TN participated in its design, coordination, carried out statistical analyses and drafted the manuscript; ER participated in the design and interpretation of the data, and helped to draft the manuscript; CR participated in the design and interpretation of the data, and helped to draft the manuscript. All authors read and approved the final manuscript.

## Pre-publication history

The pre-publication history for this paper can be accessed here:

http://www.biomedcentral.com/1471-2458/13/684/prepub
